# Inhibition of ERK downregulates autophagy via mitigating mitochondrial fragmentation to protect SH-SY5Y cells from OGD/R injury

**DOI:** 10.1186/s12964-023-01211-3

**Published:** 2023-08-14

**Authors:** Zhang-Li Yuan, Yan-Zi Mo, De-Li Li, Lu Xie, Meng-Hua Chen

**Affiliations:** 1 Department of Emergency Medicine, The Second Affiliated Hospital of Guangxi Medical University, Nanning, 530007 Guangxi People’s Republic of China; 2 Intensive Care Unit, The Second Affiliated Hospital of Guangxi Medical University, 166 Daxuedong Road, Guangxi 530007 Nanning, People’s Republic of China; 3 Guangxi Medical University, 22 Shuangyong Road, Guangxi 530021 Nanning, People’s Republic of China

**Keywords:** ERK, Drp1, Mfn2, Mitochondrial dynamics, Autophagy, OGD/R, CIRI

## Abstract

**Background:**

Cerebral ischemia-reperfusion injury (CIRI) is the main cause leading to high mortality and neurological disability in patients with cardiac arrest/cardiopulmonary resuscitation (CA/CPR). Our previous study found that extracellular signal-regulated kinase (ERK) activation, dynamin-related protein1 (Drp1)/Mitofusin2 (Mfn2)-dependent mitochondrial dynamics imbalance, and excessive autophagy were involved in the mechanism of nerve injury after CA/CPR. However, the specific pathological signaling pathway is still unknown. This study aimed to explore the molecular function changes of ERK-Drp1/Mfn2-autophagy signaling pathway in SH-SY5Y cell oxygen-glucose deprivation/reoxygenation (OGD/R) model, to further clarify the pathophysiological mechanism of CIRI, and to provide a new strategy for cerebral protection after CIRI.

**Methods:**

SH-SY5Y cells were pretreated with drugs 24 h before OGD/R. The Drp1 and Mfn2 knockdown were adopted small interfering RNAs. The overexpression of p-Drp1S616 and Mfn2 were used recombinant plasmids. The expression levels of mitochondrial dynamics proteins (p-Drp1, Drp1, Mfn2, Mfn1 and Opa1) and autophagy markers (LC3, Beclin1 and p62) were measured with the Western blotting. The mRNA levels after transfection were determined by PCR. Cell injury and viability were evaluated with released LDH activity and CCK8 assay kits. Mitochondria morphology and autophagosome were observed under transmission electron microscopy. Mitochondrial function was detected by the mitochondrial permeability transition pore assay kit. The co-expression of p-ERK, p-Drp1 and LC3 was assessed with multiple immunofluorescences. One-way analysis of variance followed by least significance difference post hoc analysis (for equal homogeneity) or Dunnett’s T3 test (for unequal homogeneity) were used for statistical tests.

**Results:**

ERK inhibitor-PD98059 (PD) protects SH-SY5Y cells from OGD/R-induced injury; while ERK activator-TPA had the opposite effect. Similar to autophagy inhibitor 3-MA, PD downregulated autophagy to improve cell viability; while autophagy activator-rapamycin further aggravated cell death. PD and Drp1-knockdown synergistically attenuated OGD/R-induced Drp1 activation, mPTP opening and cell injury; overexpression of Drp1^S616E^ or ablating Mfn2 partly abolished the protective effects of PD. Multiple immunofluorescences showed that p-ERK, p-Drp1 and LC3 were co-expressed.

**Conclusion:**

Inhibition of ERK downregulates autophagy via reducing Drp1/Mfn2-dependent mitochondrial fragmentation to antagonize mitochondrial dysfunction and promotes cell survival in the SH-SY5Y cells OGD/R model.

Video Abstract

**Supplementary Information:**

The online version contains supplementary material available at 10.1186/s12964-023-01211-3.

## Introduction

Cerebral ischemia-reperfusion injury (CIRI) is the phenomenon that the cerebral ischemia-induced brain damage is further aggravated after the recovery of blood and oxygen. CIRI predominantly contributes to the high neurological disability and mortality in patients suffering from cardiac arrest/cardiopulmonary resuscitation (CA/CPR) [1]. Unfortunately, the underlying pathological mechanism of CIRI is not fully elucidated so far. Emerging studies have shown that mitochondrial dynamics and autophagy may play an essential role in brain injury after CIRI [2, 3].

Mitochondria are remarkably dynamic organelles with constant fission/fusion to maintain their morphology, a process controlled by a series of GTPase proteins. Dynamin-related protein 1 (Drp1) is a key factor promoting mitochondrial fission [4, 5]. The activity of Drp1 is largely regulated by post-translational modifications, especially phosphorylation [6, 7]. Phosphorylation of Drp1 at serine 616 (p-Drp1 S616) promotes its translocation from cytoplasm to the surface of mitochondria, resulting in mitochondrial fission. With regards to mitochondrial fusion, Mitofusin2 (Mfn2) and Mitofusin1 (Mfn1) mediate the outer mitochondrial membrane fusion, whereas optic atrophy 1 (OPA1) drives the inner mitochondrial membrane fusion [8]. Several studies have demonstrated that mitochondrial fission/fusion imbalance is implicated in CIRI-induced neuron injury. Guo et al. [9] suggested that the phosphorylation of Drp1 is increased in hippocampal HT22 neurons after oxygen glucose deprivation/reoxygenation (OGD/R) and promotes mitochondrial fission, while inhibition of Drp1-dependent mitochondrial fission alleviates neuronal injury. Chen et al. [10] demonstrated that OGD/R induces degradation of Mfn2 in reperfusion, while overexpression of Mfn2 ameliorates mitochondrial fragmentation and neuron injury. Excessive mitochondrial fission is a prominent early upstream event in neuron death, and this is probably related with autophagy [11].

Autophagy is a highly conserved mechanism maintaining intracellular homeostasis under physiological and pathological conditions. Accumulating studies [3, 12] have shown that autophagy can be activated in CIRI and may be a double-edged sword to neuron survival/death. Some evidences have shown that autophagy has neuroprotective effects and improves the prognosis of CIRI [13, 14]. However, plenty studies demonstrated that type II programmed cell death induced by excessive autophagy aggravates brain damage [15–17]. Our previous study [18] observed that mitochondrial fission was activated, accompanied by autophagy upregulation in the rat cerebral cortex following CA/CPR. However, how mitochondrial dynamics and autophagy are involved in CIRI remains elusive.

The extracellular signal-regulated kinase (ERK), a member of the mitogen-activated protein kinase (MAPK) family, takes part in various biological reactions by regulating numerous nuclear transcription factors and cytoplasmic proteins. Our previous studies [18, 19] have found that ERK is significantly activated in the rat cerebral cortex after CA/CPR. ERK inhibitor-PD98059 (PD) ameliorates brain damage accompanied by downregulating Drp1/Mfn2-dependent mitochondrial fission and autophagy, but the specific molecular mechanisms have yet to be fully illustrated.

Upon the studies mentioned above, we hypothesized that inhibition of ERK downregulates autophagy via ameliorating Drp1/Mfn2-dependent mitochondrial fragmentation to protect SH-SY5Y cells from OGD/R injury. To verify this hypothesis, we used OGD/R-treated SH-SY5Y cells in vitro to mimic CIRI; then we tested the roles of ERK and autophagy in OGD/R-induced injury, examined the effect of ERK on autophagy; finally, we applied knockdown/overexpression of Drp1 and Mfn2 to determine whether inhibition of ERK downregulates autophagy via Drp1/Mfn2 to mitigate OGD/R-induced neuronal injury. This study further systematically elaborated on the pathophysiological mechanism of CIRI and provided a new strategy for the brain protection.

## Materials and methods

### Cell culture and reagents

SH-SY5Y cell line was purchased from China Center for Type Culture Collection (CCTCC, China) and cultured in normal medium [MEM (11,140,050; Gibco, U.S.):F12 (21,700,075; Gibco, U.S.) = 1:1, supplemented with 10% fetal bovine serum (10,100,147; FBS; Gibco, U.S.), 1% penicillin and 1% streptomycin]. The cells were preserved at 37℃ in a humidified incubator with 5% CO_2_. The reagents used in this experiment included PD98059 (167869-21-8; Sigma-Aldrich, U.S.), dimethyl sulfoxide (67-68-5; DMSO; Sigma-Aldrich, U.S.), 12-*O*-tetradecanoylphorbol-13-acetate (TPA; N2060; ApexBio, U.S.), rapamycin (A8167; ApexBio, U.S.), and 3-methyladenine (3-MA; A8353; ApexBio, U.S.).

### Oxygen and glucose deprivation/reoxygenation (OGD/R) model and experimental groups

SH-SY5Y cells were washed with PBS and incubated in glucose-free DMEM (11,966,025; Gibco, U.S.) in an anaerobic container to initiate OGD insult for 2 h. Then the DMEM was replaced with normal medium. The cells were returned to the incubator with 5% CO_2_ to initiate reoxygenation for 24 h.

To exam the roles of ERK in OGD/R-induced injury, to evaluate the effects of autophagy on OGD/R-induced injury and to explore whether inhibition of ERK mitigates autophagy via inactivating Drp1, cells were assigned to different group sets (see Results section below).

### Cell transfection

Mfn2 and Drp1 si-RNA were ordered from Sangon Biotech. Mfn2 and Drp1 recombinant plasmids were ordered from GeneCopoeia. Both si-RNA and recombinant plasmids were delivered to SH-SY5Y cells using Lipofectamine-3000 reagent (L3000015; Invitrogen, U.S.) according to the manufacturer’s protocol. SH-SY5Y cells were seeded in 6-well plates. Twenty-four hours later, si-RNA or recombinant plasmids mixed with Lipo-3000 reagent were added into Opti-MEM (31,985,070; Gibco, U.S.), then the Opti-MEM was added into the wells for transfection. Twenty-four hours later, the Opti-MEM was replaced with normal medium to stop transfection. In this experiment, synthetic Drp1S616E was used as a model to simulate p-Drp1S616. Serine 616 (S) of Drp1 is a phosphorylation site of the protein. When the site-specific mutation from Serine (S) bonds to the negatively charged glutamic acid (E), steric hindrance and charge of this site are similar to those of the phosphate group (PO_4_^3−^), thus it may simulate the phosphorylation of Drp1S616 (p-Drp1S616) and induce the subsequent effect of p-Drp1S616. This technology has been widely used in biomedical research [20, 21].

### Western blot

SH-SY5Y cells were lysed with RIPA Lysis Buffer (P0013B; Beyotime, China). The concentration of total protein was measured by protein assay kit (AR0146; Boster, China). The protein samples were separated using 10% or 12% SDS-PAGE gel and blotted to PVDF membranes. Subsequently, the PVDF membranes were incubated overnight at 4℃ in primary antibodies as follows: p-ERK (1:1000; #4370, CST, U.S.), ERK (1:1000; ab184699, Abcam, U.K.), p-Drp1S616 (1:1000; #3455, CST, U.S.), Drp1 (1:1000; #8570S, CST, U.S.), Mfn2 (1:1000; #9482S, CST, U.S.), Mfn1 (1:1000; #14,739, CST, U.S.), Opa1 (1:1000; #67589S, CST, U.S.), LC3B (1:2000; ab192890, Abcam, U.K.), Beclin1 (1:2000; 11,306, Proteintech, China), p62 (1:1000; #5114, CST, U.S.), GAPDH (1:1000; #5174, CST, U.S.). After being washed with Tris-buffered saline, the membranes were incubated in anti-rabbit horseradish peroxidase-conjugated antibodies (1:10000; Santa Cruz Biotechnology, U.S.) for 1 h at room temperature. The target protein bands were detected with Odyssey imaging system (LI-COR, U.S.).

### Real time polymerase chain reaction (RT-PCR)

RNA was extracted with NucleoZol (740404.200; MNG, German). Reverse transcription to cDNA was performed with PrimeScript™ RT Master Mix (RR036A; Takara, Japan) and qPCR was performed with SYBR Premix Ex Taq™ II (RR820A; Takara, Japan) according to manufacturer’s instructions. The primer sequences of Drp1 are as follows: forward 5’-AGGAGAAGAAAATGGGGTGG-3’, Reverse 5’-ACCGAAGAATGAGCTCTCTG-3’. The mRNA expression of Drp1 was normalized to that of GAPDH. The results were calculated by the 2^−ΔΔCt^ method.

### Transmission electron microscopy (TEM) observation

SH-SY5Y cells were collected in a tube after centrifugation and preserved in 4% para-formaldehyde. Then the fixed cells were washed with phosphate buffer and immersed in 1% agarose solution. Subsequently, the agarose blocks with samples were fixed with 1% OsO_4_, dehydrated in ethanol and embedded in epoxy resin. The resin blocks were cut into 80 nm thin slices, which were double stained with acetate and lead citrate. The samples were observed under TEM (HT7800, Hitachi, Japan).

### Mitochondrial permeability transition pore (mPTP) opening measurement

MPTP opening was measured with mPTP Assay Kit (C2009S; Beyotime, China) according to the manufacturer’s instruction. SH-SY5Y cells were seeded in 24-well plates. The cells were immersed in fluorescence quenching solution for 40 min, then the solution was replaced with normal medium in the dark. Thirty minutes later, changed the normal medium to assay buffer. The fluorescently labeled cells were observed under fluorescence microscope.

### Immunofluorescence detection

The objective area of cell climbing slides were incubated with permeabilization working solution for 20 min, then covered with 3% BSA for 30 min. Subsequently, incubate cells with primary antibodies included anti-p-ERK (1:400; #4370, CST), anti-p-Drp1 (1:400; #3455, CST) and anti-LC3 (1:1000; ab192890, Abcam) overnight at 4℃. Next, the slides were washed and incubated with horseradish peroxidase-conjugated secondary antibodies responding to primary antibody in species for 50 min. The cell climbing slides were incubated in DAPI solution for 10 min in the dark and observed under fluorescent microscopy (p-ERK-positive cells were observed green in FITC channel; p-Drp1-positive cells were observed pink in CY5 channel; LC3-positive cells were observed red in CY3 channel).

### Lactate dehydrogenase (LDH) activity assay

The activity of LDH released from cells was measured by LDH activity assay kit (A020-2; Nanjing Jiancheng, China) following the manufacturer’s protocol to assess cell injury. The cell medium was collected to 48-well plates. The required solution was prepared according to the instructions and added into the cell medium. The absorbance at 450 nm was detected by microplate reader (680; Bio-Rad, U.S.).

### Cell counting kit-8 (CCK-8) assay

Cell viability were measured by CCK-8 (C6005; NCM Biotech, U.S.) following the manufacturer’s specifications. Briefly, SH-SY5Y cells were seeded in 96-well plates. CCK-8 solution was added to the wells with 10% concentration. The cells were incubated at 37℃. Two hours later, the absorbance at 450 nm was detected by microplate reader (680; Bio-Rad, U.S.).

### ATP content assay

The intracellular ATP content were measured with a luminescence-based ATP assay kit (S0026, Beyotime, China) according to the manufacturer’s specifications. Cells were lysed. The supernatants were acquired after centrifugation and mixed with prepared ATP detection buffer. Luminescence signals was measured by multifunctional enzyme labelling instrument (iMark, Bio-Rad, U.S.).

### Statistical analysis

Statistical analyses were performed with SPSS 21.0 software (IBM, U.S.). The Shapiri-Wilk test was conducted to evaluate normality of the data. The normally distributed data were presented as mean ± standard deviation (SD). One-way analysis of variance (ANOVA) followed by least significance difference (LSD) post hoc analysis (for equal homogeneity) or Dunnett’s T3 test (for unequal homogeneity) were used to assess the differences between groups. Nonparametric tests were used to estimate the non-normally distributed data. *P* < 0.05 was regarded statistically significant.

## Results

### Inhibition of ERK protects SH-SY5Y cells from OGD/R-induced injury

**Fig. 1:**
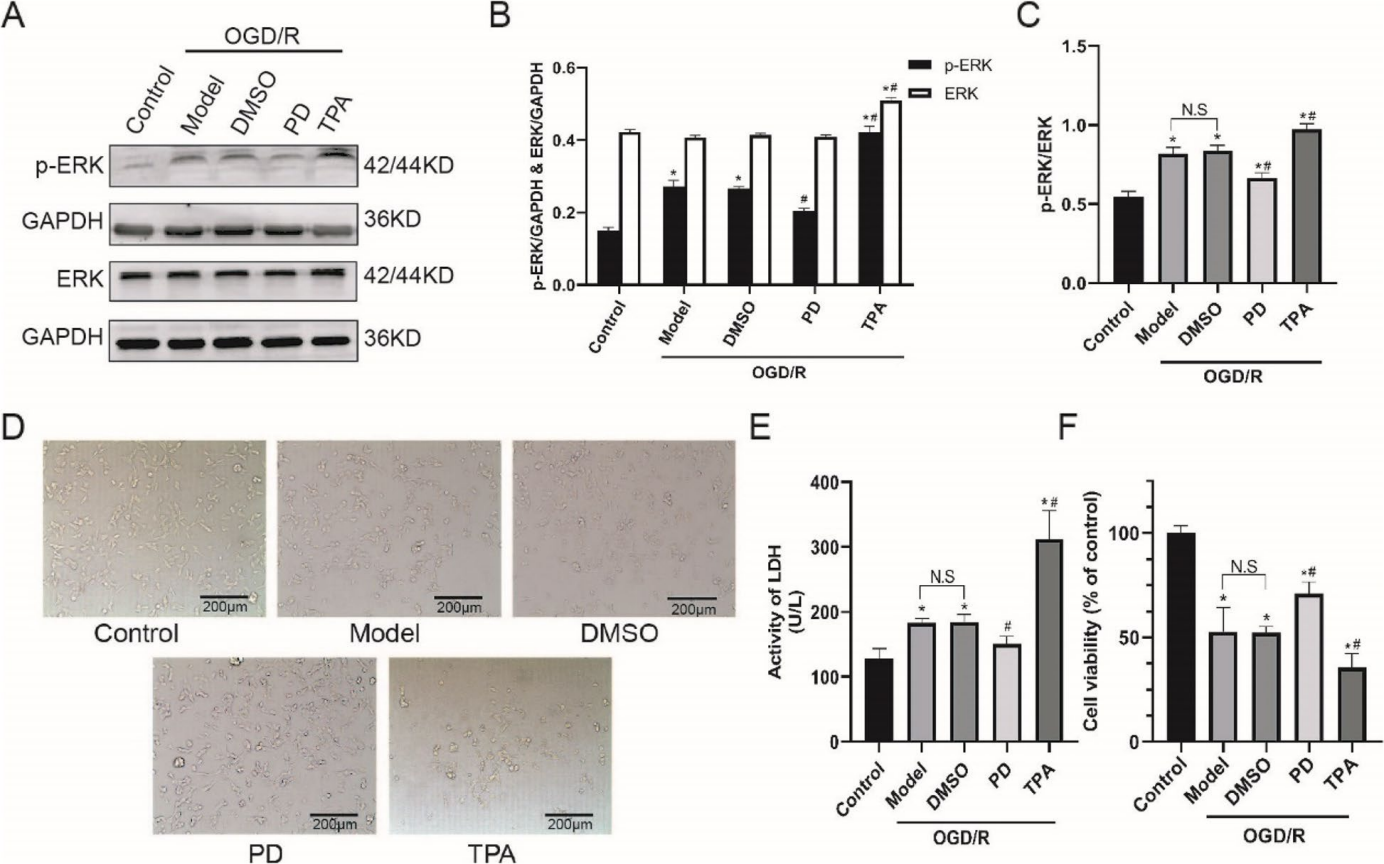
Inhibition of ERK protects SH-SY5Y cells from OGD/R-induced injury

In this experiment, PD was used to inhibit ERK activation, and TPA was used to activate ERK. As expected, PD antagonized OGD/R-triggered increment of p-ERK/ERK (*P* < 0.05), while TPA further increased the ratio of p-ERK/ERK (*P* < 0.05) (Fig. 1-A ~ C). Next, we detected the effects of ERK on cell morphology, injury and cell viability under OGD/R. In Fig. 1D, a significant loss of cells and synapses were observed after OGD/R; PD alleviated cells and synapses loss; conversely, TPA induced the opposite effects. In Fig. 1E, PD attenuated OGD/R-induced cell injury as suggested by lower LDH activity (*P* < 0.05), while TPA further aggravated cell injury (*P* < 0.05). In Fig. 1F, PD increased cell viability and protected the cells against OGD/R-induced death (*P* < 0.05); conversely, TPA decreased cell viability and aggravated cell death (*P* < 0.05).

A, Representative immunoblot bands of p-ERK and ERK; B-C, Quantitative analysis of p-ERK, ERK and p-ERK/ERK; D, Morphology of cells under optical light microscope, the scale bar: 200 μm; E-F, LDH and CCK-8 assays were performed to detect cell injury and viability. All data in this figure are representative of three independent repeats and are shown as mean ±SEM. DMSO: solvent control group; PD: PD98059 (ERK inhibitor) group; TPA: 12-*O*-tetradecanoylphorbol 13-acetate (ERK activator). *P < 0.05 vs. Control; #*P* < 0.05 vs. DMSO.

### Downregulation of autophagy by ERK inhibition alleviates OGD/R-induced injury in SH-SY5Y cells

**Fig. 2:**
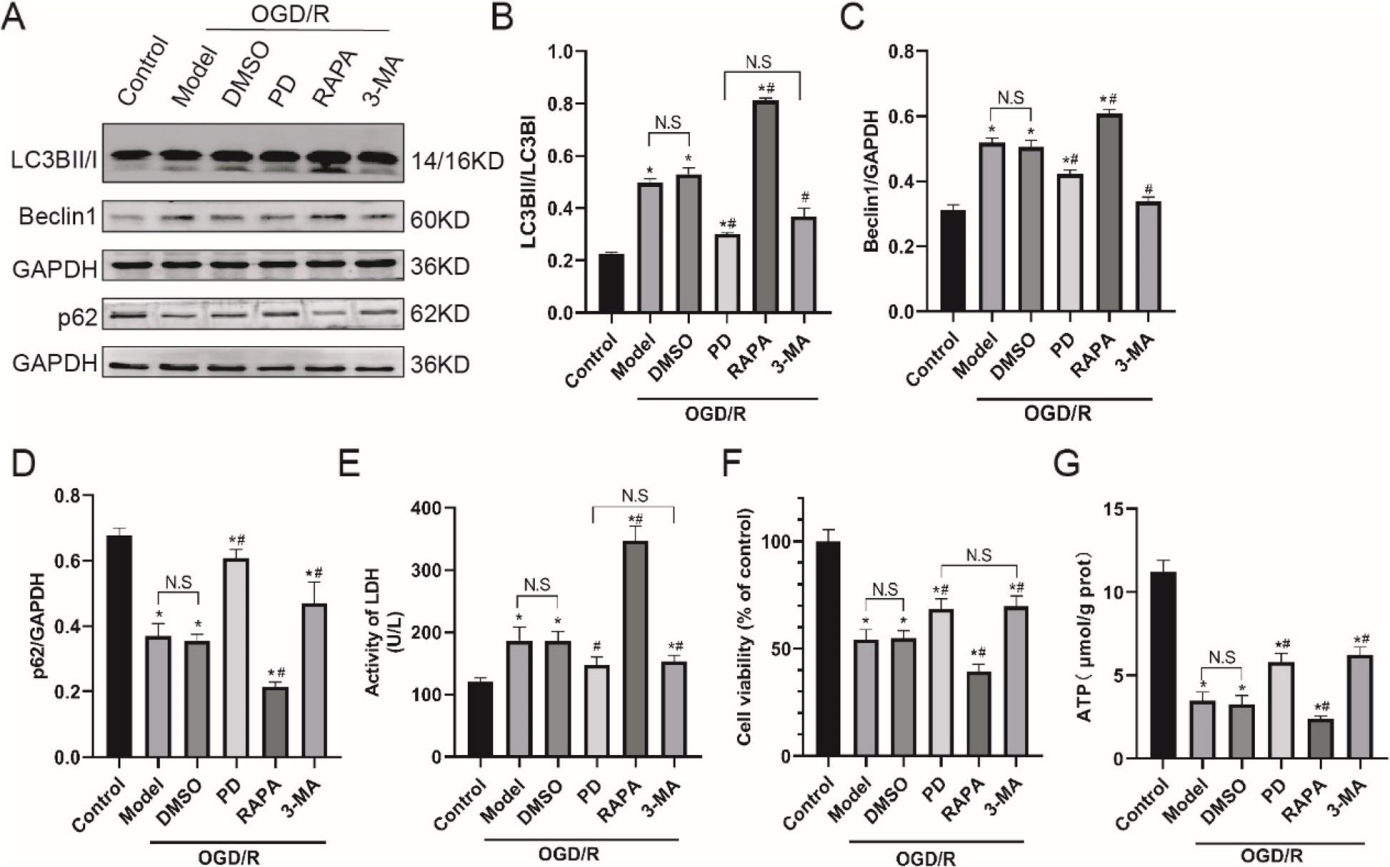
Downregulation of autophagy by ERK inhibition alleviates OGD/R-induced injury and death in SH-SY5Y cells

To address the role of ERK inhibition in autophagy, we tested the effect of PD on autophagic proteins (Fig. 2A). In Fig. 2B ~ D, similar to 3-MA, PD downregulated the expression levels of LC3BII/I and Beclin1, increased p62 cumulation (*P* < 0.05), suggesting that inhibition of ERK decreases OGD/R-triggered autophagy activation. Then, the effect of autophagy on cell injury and viability after OGD/R was detected. In Fig. 2E and -F, PD and 3-MA mitigated cell injury and death as indicated by lower released LDH activity and higher cell viability (*P* < 0.05); while rapamycin presented opposite effects (*P* < 0.05). In Fig. 2G, PD and 3-MA partly restored intracellular ATP content after OGD/R, while rapamycin aggravated ATP content. Taken together, these results suggested that inhibition of ERK downregulates autophagy to attenuate OGD/R-induced injury and death in SH-SY5Y cells.

A, Representative immunoblot bands of LC3B, Beclin1 and p62; B-D, Quantitative analysis of LC3B, Beclin1 and p62; E-F, LDH and CCK-8 assays were performed to detect cell injury and viability; G, ATP assay were tested to evaluate mitochondrial function. All data in this figure are representative of three independent repeats and are shown as mean ±SEM. DMSO: solvent control group; PD: PD98059 (ERK inhibitor) group; Rapa: Rapamycin (autophagy stimulator); 3-MA: 3-methyladenine (autophagy inhibitor). *P < 0.05 vs. Control; #*P* < 0.05 vs. DMSO.

### The efficiency of knockdown/overexpression of Drp1 and Mfn2

**Fig. 3:**
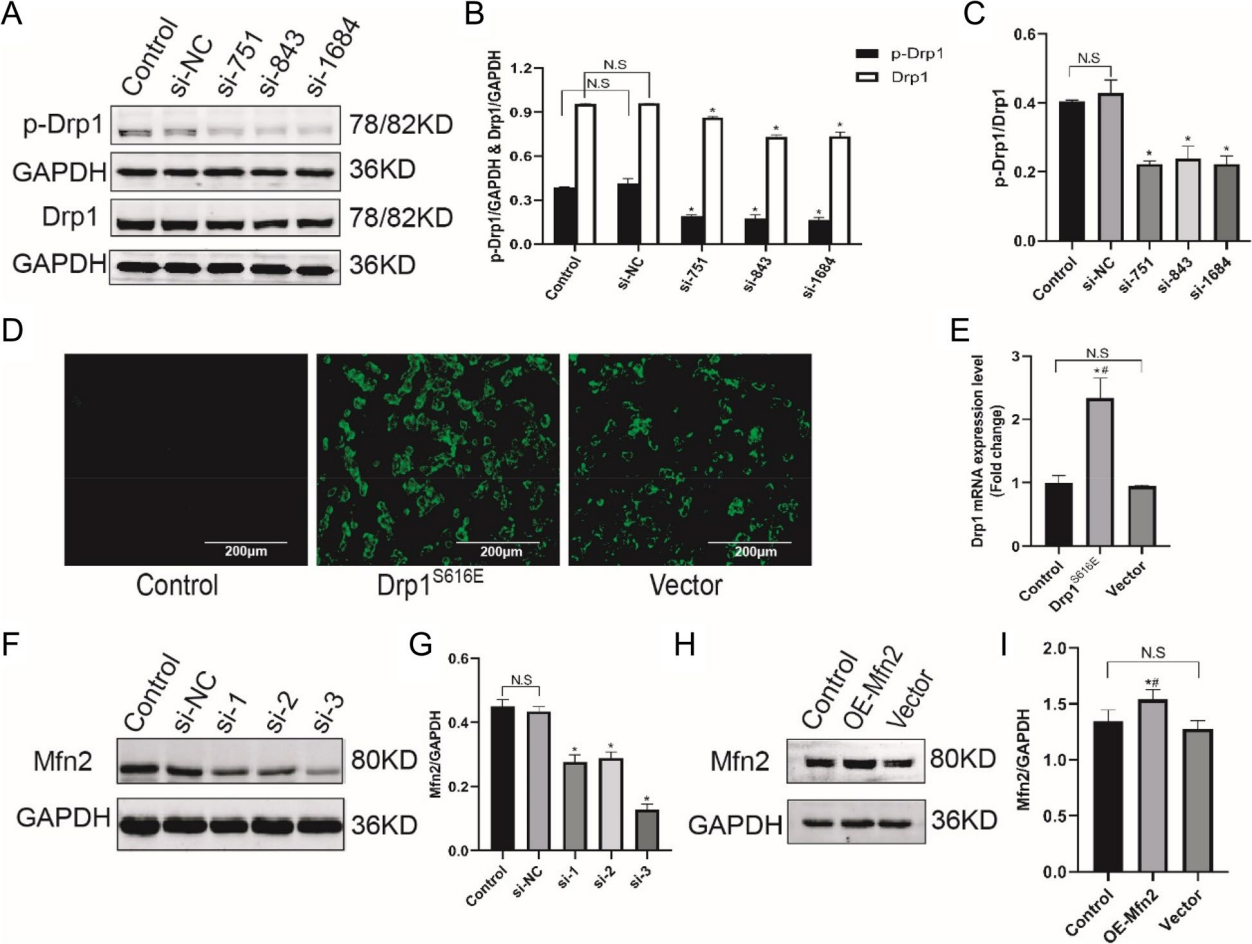
Transfection efficiency of si-RNA and overexpression plasmid. **A** Representative immunoblot bands of p-Drp1 and Drp1; **B**, **C**, Quantitative analysis of p-Drp1 and Drp1; **D**, SH-SY5Y cells transfected with Drp1^S616E^-GFP-M90 plasmid after 24 h observed under fluorescence microscope; **E**, Quantitative analysis of fluorescence intensity; **F**, **H**, Representative immunoblot bands of Mfn2 after transfected with si-Mfn2 and Mfn2 pcDNA3.1; **G**, **I**, Quantitative analysis of Mfn2. All data in this figure are representative of three independent repeats and are shown as mean ±SEM. si-NC: Drp1 si-RNA negative control group; si-751/si-843/si1684: Drp1 si-RNA sequences; Drp1^S616E^: Drp1^S616E^-GFP-M90 plasmid; Vector: Drp1^S616E^ negative control group; si-1/si-2/si-3: Mfn2 si-RNA sequences; OE-Mfn2: Mfn2 pcDNA3.1. *P < 0.05 vs. si-NC and Control; #*P* < 0.05 vs. Vector

To explore whether Drp1/Mfn2-dependent mitochondrial dynamics is involved in the downregulation of autophagy by PD, Drp1 and Mfn2 knockdown/overexpression in SH-SY5Y cells were performed. The efficiency of transfection was verified. In Fig. 3A ~ C, compared with the control group, all three Drp1 si-RNAs inhibited the expression of Drp1 and p-Drp1 proteins (*P* < 0.05); interestingly, we found that knockdown of Drp1 also decreased the ratio of p-Drp1/Drp1. In Fig. 3D, Synthetic Drp1^S616E^ was transfected with M90 plasmid carrying GFP green fluorescent tag to mimic Drp1S616 activation. Green fluorescence was almost invisible in the control group but was obvious in transfection groups. In Fig. 3E, RT-PCR showed that Drp1^S616E^ transfection increased the expression of Drp1 mRNA by more than 50% (*P* < 0.05). In Fig. 3F, G, Mfn2 si-RNA induced a decrement of Mfn2 protein (*P* < 0.05). In Fig. 3H, I, over expression of Mfn2 (OE-Mfn2) increased the expression of Mfn2 but the transfection efficiency was less than 50% compared with the vector group.

### Inhibition of ERK alleviates Drp1-dependent mitochondrial fission and injury after OGD/R in SH-SY5Y cells

**Fig. 4:**
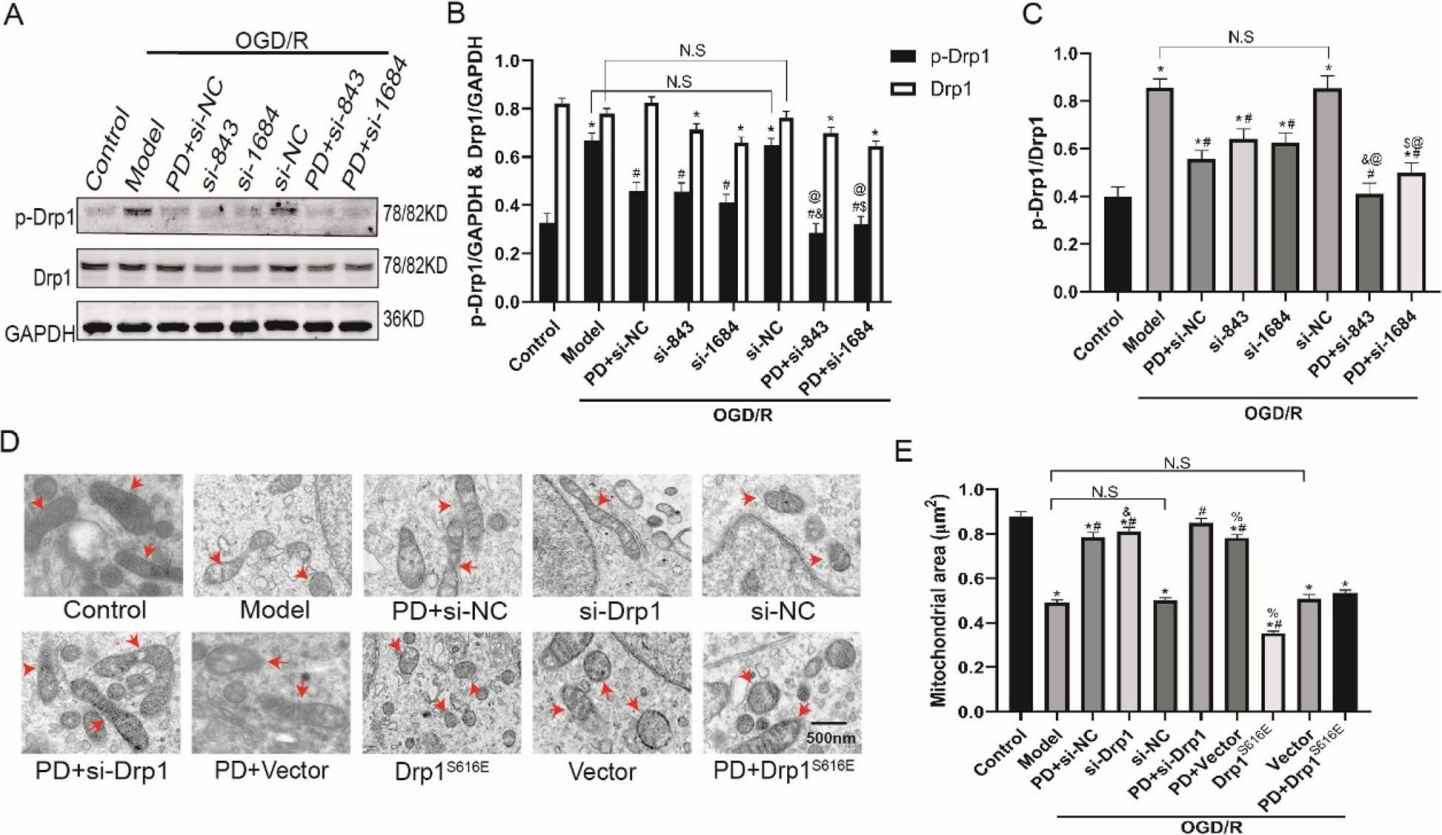
Inhibition of ERK alleviates p-Drp1S616 dependent mitochondrial fission and injury after OGD/R in SH-SY5Y cells. **A** Representative immunoblot bands of p-Drp1 and Drp1; **B**, **C**, Quantitative analysis of p-Drp1 and Drp1; **D**, Transmission electron microscopy images of mitochondria, the red arrows shows the mitochondria; **E**, Quantitative analysis of mitochondrial area. All data in this figure are representative of three independent repeats and are shown as mean ±SEM. si-NC: Drp1 si-RNA negative control group; si-751/si-843/si1684: Drp1 si-RNA sequences; Drp1^S616E^: Drp1^S616E^-GFP-M90 plasmid; Vector: Drp1^S616E^ negative control group. *P < 0.05 vs. Control, #*P* < 0.05 vs. si-NC, &*P* < 0.05 vs. si-843, $*P* < 0.05 vs. si-1684, @*P* < 0.05 vs. PD + si-NC

To illuminate the effect of ERK inhibition on Drp1-dependent mitochondrial dynamics, we performed WB to measure p-Drp1S616/Drp1 (Fig. 4A) and observed mitochondria under transmission electron microscope (Fig. 4D). As shown in Fig. 4B, C, PD and Drp1 si-RNA decreased the ratio of p-Drp1S616/Drp1 (*P* < 0.05); moreover, the decline of p-Drp1S616/Drp1 in the combination group was greater (*P* < 0.05), suggesting that inhibition of ERK and ablating Drp1 synergistically attenuates OGD/R-induced Drp1 activation. In Fig. 4D, inhibition of ERK and ablating Drp1 protected mitochondria from OGD/R injury, as indicated by less severe mitochondrial swelling and internal vacuolization. In Fig. 4E, the average mitochondrial area was calculated. PD and Drp1 si-RNA reduced the mitochondrial fission induced by OGD/R (*P* < 0.05); in addition, the mitochondrial area in the combination group was even larger than that in the single group (*P* < 0.05). Of note, Drp1^S616E^ overexpression not only aggravated mitochondrial swelling, internal vacuolization and mitochondrial fission, but also abolished the protective effect to mitochondria of PD (Fig. 4D, E). Collectively, these results illuminated that inhibition of ERK alleviates p-Drp1S616-dependent mitochondrial fission, at least partly, to protects mitochondria from OGD/R injury.

### Inhibition of ERK downregulates autophagy by reducing Drp1-dependent mitochondrial fission after OGD/R in SH-SY5Y cells

**Fig. 5:**
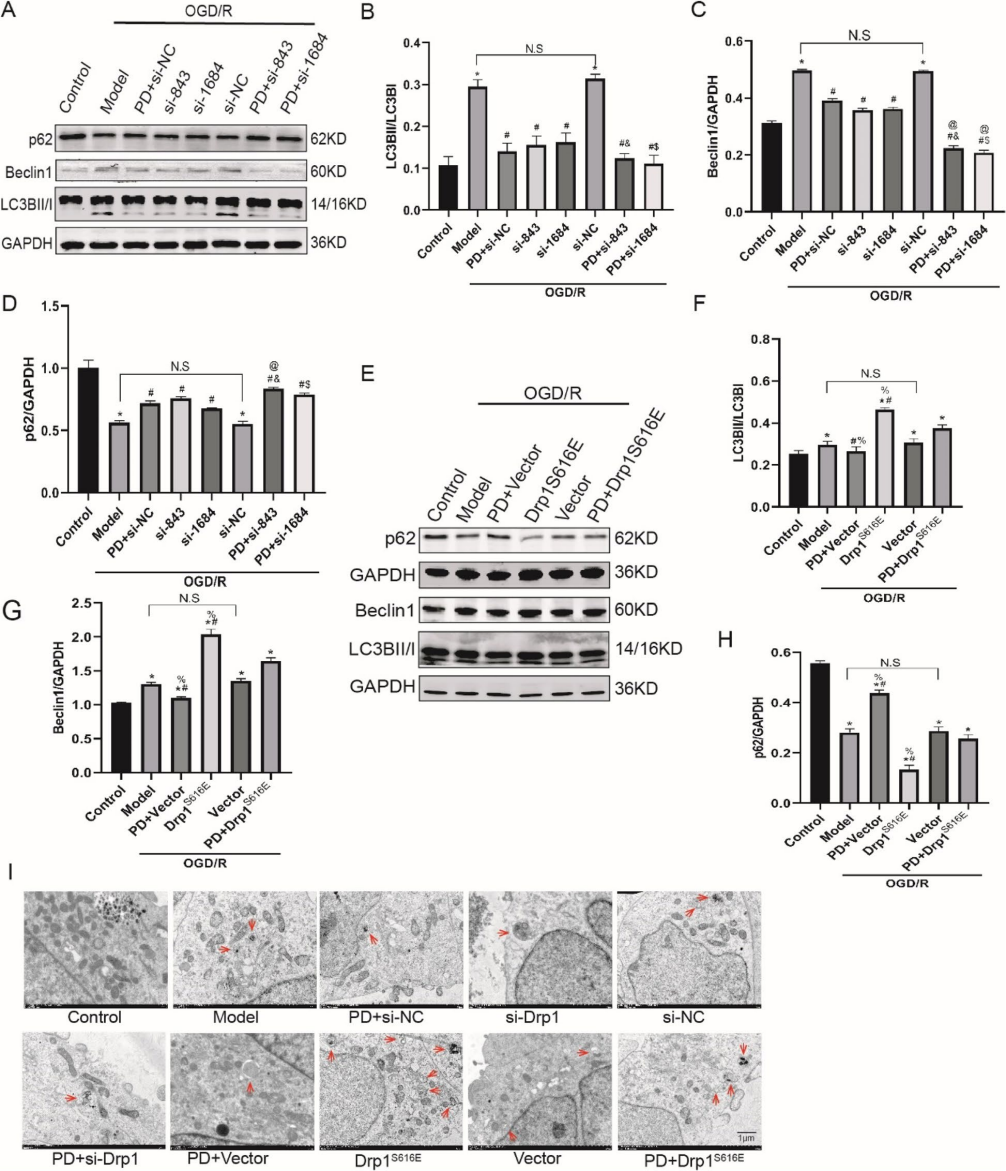
Inhibition of ERK downregulates autophagy by reducing Drp1-dependent mitochondrial fission after OGD/R in SH-SY5Y cells. **A**, **E**, Representative immunoblot bands of LC3B, Beclin1 and p62; **B**-**D**, **F**-**H**, Quantitative analysis of LC3B, Beclin1 and p62; I. Transmission electron microscopy images of autophagosome/autolysosome (red arrows). All data in this figure are representative of three independent repeats and are shown as mean ±SEM. si-NC: Drp1 si-RNA negative control group; si-751/si-843/si1684: Drp1 si-RNA sequences; Drp1^S616E^: Drp1^S616E^-GFP-M90 plasmid; Vector: Drp1^S616E^ negative control group. *P < 0.05 vs. Control, #*P* < 0.05 vs. si-NC, &*P* < 0.05 vs. si-843, $*P* < 0.05 vs. si-1684, @*P* < 0.05 vs. PD + si-NC

To explore the mechanism of ERK-Drp1-autophagy pathway in OGD/R-treated cells, we first observed the effect of Drp1 si-RNA on autophagy (Fig. 5A). In Fig. 5B ~ D, PD and Drp1 si-RNA attenuated OGD/R-induced increment in LC3BII/I and Beclin1 expression, and promoted p62 cumulation (*P* < 0.05); additionally, the combination group presented greater variation (*P* < 0.05). Then we observed overexpression of Drp1^S616E^ partially reversed the downregulation of autophagy by PD (*P* < 0.05) (Fig. 5E ~ H). The changes of autophagosomes/autolysosomes in different groups were consistent with the expression levels of LC3, Beclin1 and p62. Taken together, these results evidenced that inhibition of ERK downregulates autophagy, at least partly, via reducing p-Drp1S616-dependent mitochondrial fission in OGD/R-treated SH-SY5Y cells.

### Inhibition of ERK prevents mPTP opening and protects from OGD/R injury by reducing Drp1 activation in SH-SY5Y cells

**Fig. 6:**
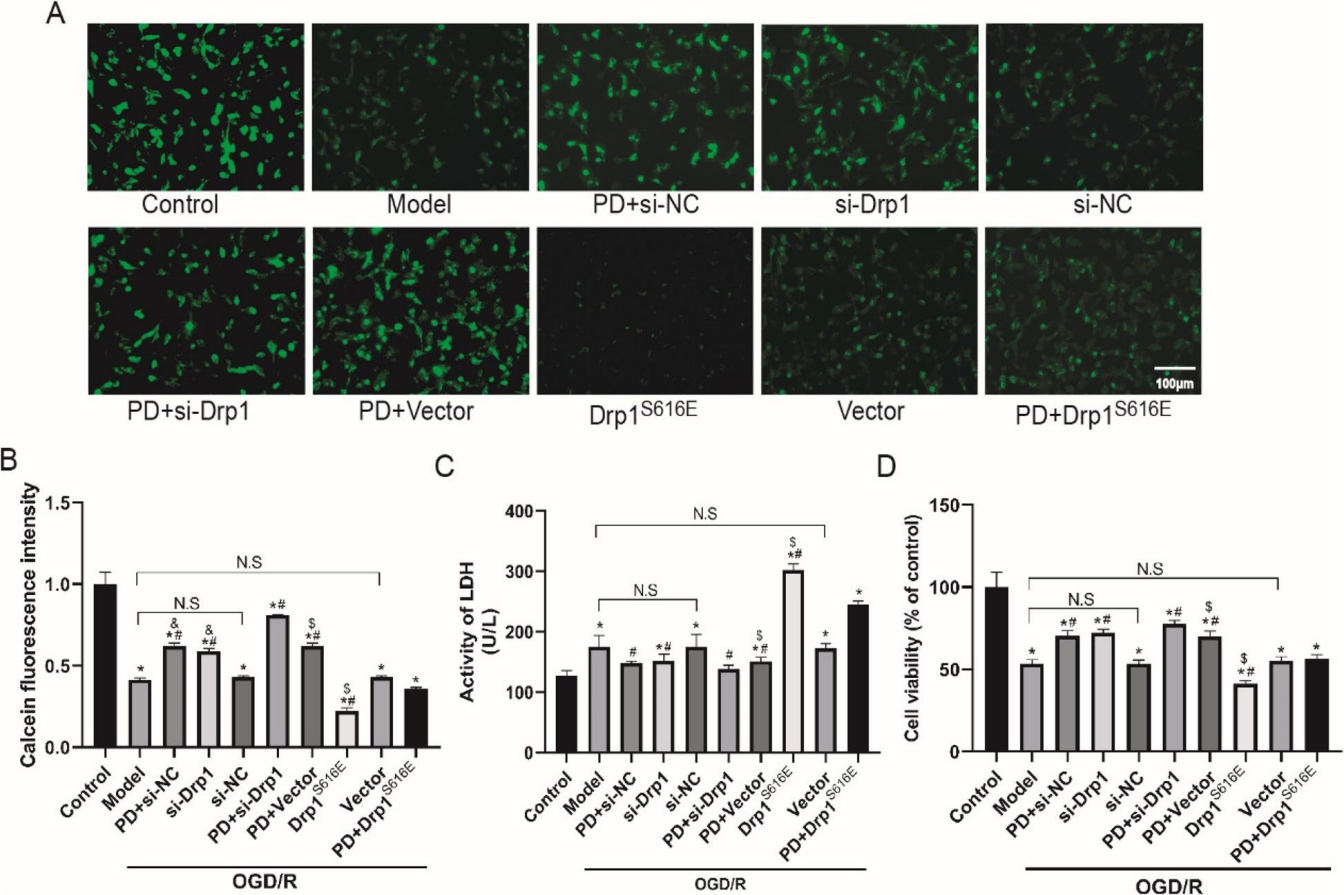
Inhibition of ERK prevents mPTP opening and protects from OGD/R injury by reducing Drp1 activation in SH-SY5Y cells. **A** The opening of mPTP was detected by loading with calcein AM (green fluorescence). The intensity of green fluorescence was negatively correlated with the opening of mPTP, scare bar: 100 μm. **B** Quantitative analysis of calcein AM florescence intensity. **C**, **D**, LDH and CCK-8 assays were performed to detect cell injury and viability. All data in this figure are representative of three independent repeats and are shown as mean ±SEM. si-NC: Drp1 si-RNA negative control group; si-Drp1: Drp1 si-RNA sequences; Drp1^S616E^: Drp1^S616E^-GFP-M90 plasmid; Vector: Drp1^S616E^ negative control group. *P < 0.05 vs. Control, #*P* < 0.05 vs. Model, &*P* < 0.05 vs. si-NC, $*P* < 0.05 vs. Vector

To elucidate the role of Drp1 in OGD/R-induced mitochondrial dysfunction and injury, we tested the opening level of mPTP, LDH release activity and cell viability upon Drp1 knockdown/overexpression. In Fig. 6A, the intensity of Calcein-AM (green fluorescence) was negatively correlated to the opening degree of mPTP. In Fig. 6B, the OGD/R model group exhibited dimmer fluorescence intensity compared with the control group (*P* < 0.05), suggesting that OGD/R induced the opening of mPTP; the fluorescence intensity in the PD and Drp1 si-RNA enhanced compared with the OGD/R model group, the PD + Drp1 si-RNA group presented stronger florescence intensity than the single groups (*P* < 0.05); conversely, the fluorescence intensity in Drp1^S616E^ overexpression group was the weakest (*P* < 0.05); Drp1^S616E^ overexpression restored the PD-suppressed opening level of mPTP (*P* < 0.05). These results demonstrated that inhibition of ERK improves mitochondrial dysfunction partly via inactivating Drp1 after OGD/R. In Fig. 6C, compared with the OGD/R model group, LDH activity in the PD, Drp1 si-RNA and PD + Drp1 si-RNA group reduced (*P* < 0.05). On the contrary, LDH activity in the Drp1^S616E^ overexpression group increased (*P* < 0.05); in addition, Drp1^S616E^ overexpression abolished the inhibitory effect on LDH by PD (*P* < 0.05). In Fig. 6D, PD and Drp1 si-RNA restored cell viability in the OGD/R-treated cells (*P* < 0.05); notably, the protection of PD was partly blunted by Drp1^S616E^ overexpression (*P* < 0.05), indicating that inhibition of ERK protects from OGD/R injury by reducing p-Drp1S616 in SH-SY5Y cells.

### The co-expression of p-ERK, p-Drp1 and LC3II in OGD/R-treated SH-SY5Y cells

**Fig. 7:**
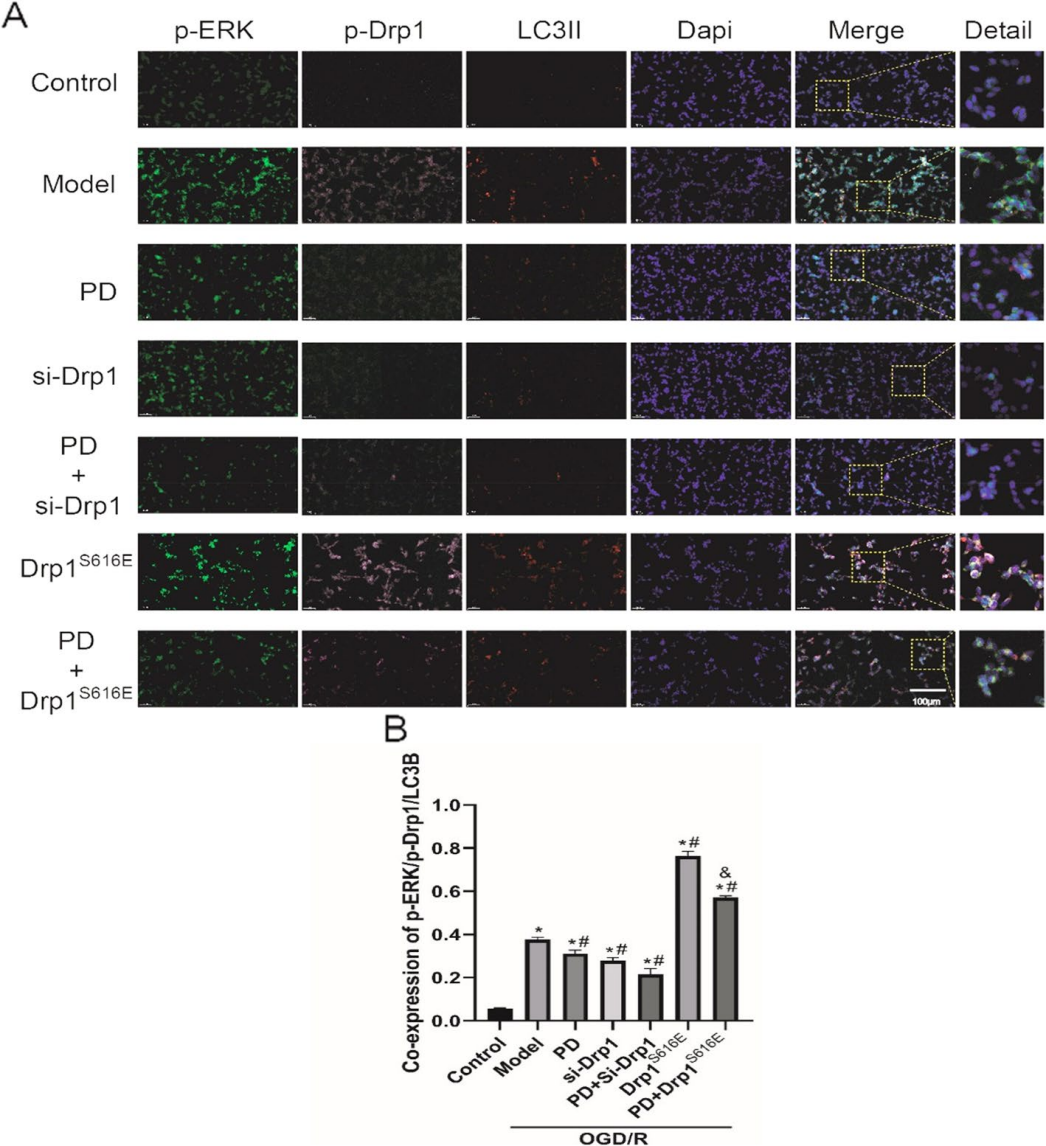
p-ERK, p-Drp1 and LC3II are co-expressed in OGD/R-treated SH-SY5Y cells. **A** Multiple immunofluorescence images of p-ERK (green), p-Drp1 (pink) and LC3II (red) proteins at 24 h after OGD/R. Nuclei were stained with Dapi (blue), scare bar: 100 μm. Detail represents the enlarged view of the rectangles in Merge. **B** Quantitative analysis of p-ERK + p-Drp1 + LC3II positive cell rate. All data in this figure are representative of three independent repeats and are shown as mean ±SEM. PD: PD98059 group; si-Drp1: Drp1 si-RNA sequences; Drp1^S616E^: Drp1^S616E^-GFP-M90 plasmid; *P < 0.05 vs. Control, #*P* < 0.05 vs. Model, &*P* < 0.05 vs. Drp1^S616E^

Based on the above results, we suggested that ERK-Drp1-autophagy pathway was involved in OGD/R-induced injury, which was reconfirmed by multiple immunofluorescence staining of p-ERK, p-Drp1 and LC3II (Fig. 7A). In Fig. 7B, PD antagonized OGD/R-triggered increase in co-expression rate of p-ERK, p-Drp1 and LC3II (*P* < 0.05). While the co-expression rate in the overexpressed Drp1^S616E^ group further increased (*P* < 0.05), Drp1 ^S616E^ overexpression reversed the decrement of co-expression by PD (*P* < 0.05). These results reconfirmed that ERK-Drp1-autophagy pathway was involved in the pathological mechanism in OGD/R-insulted SH-SY5Y cells.

### The expression of p-ERK, p-Drp1 and LC3II at different time points of OGD/R

**Fig. 8:**
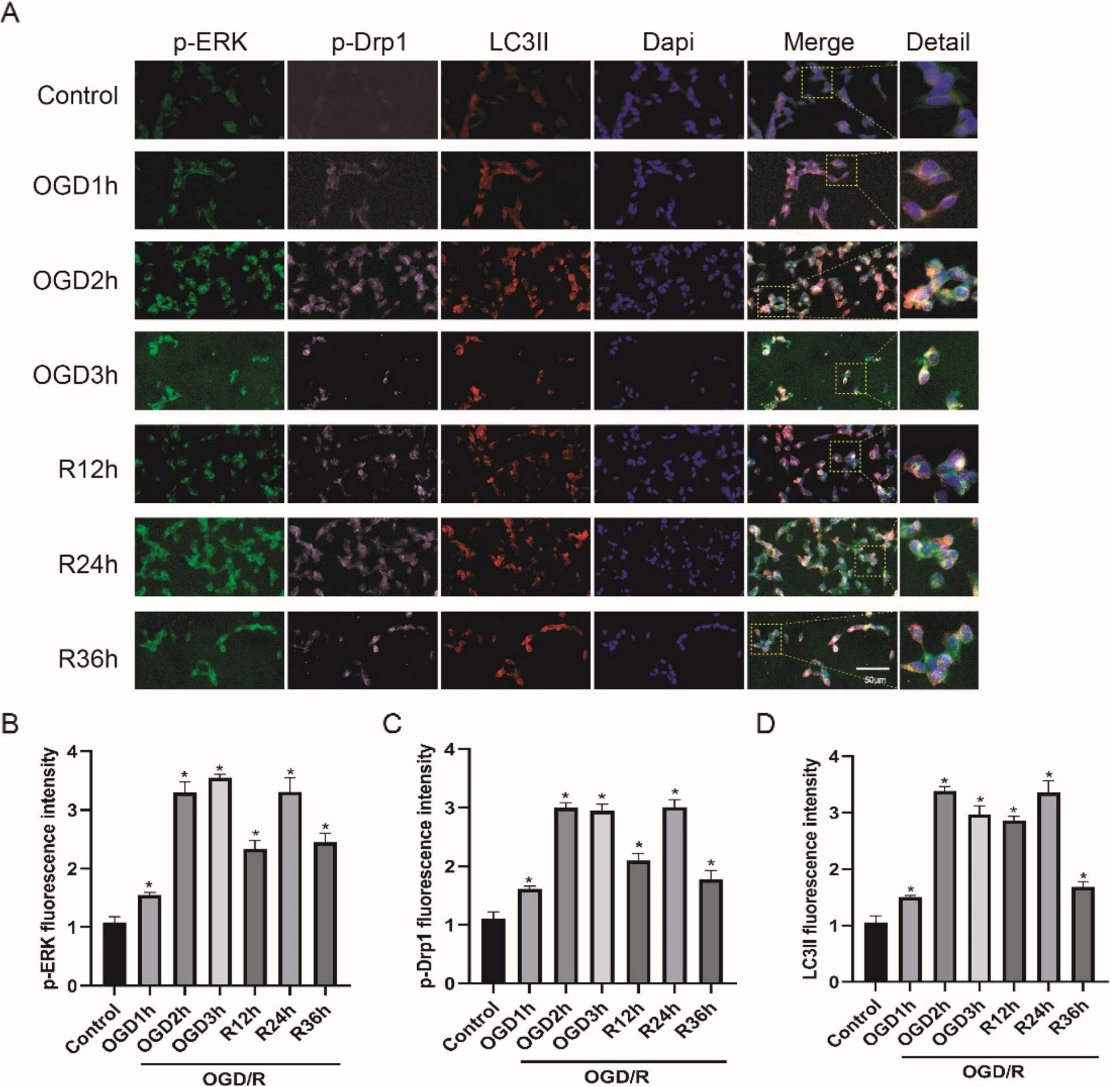
The expression of p-ERK, p-Drp1 and LC3II at different time points of OGD/R. **A** Multiple immunofluorescence images of p-ERK (green), p-Drp1 (pink) and LC3II (red) proteins at OGD1h/2 h/3 h reoxygenation 24 h, and OGD2h reoxygenation 12 h/24 hrs/36 hrs. Nuclei were stained with Dapi (blue), scare bar: 50 μm. Detail represents the enlarged view of the rectangles in Merge. **B**-**D**, Quantitative analysis of p-ERK, p-Drp1 and LC3II expression. All data in this figure are representative of three independent repeats and are shown as mean ±SEM. OGD1h/2 h/3 h: oxygen-glucose deprivation 1 h/2 hrs/3 hrs and reoxygenation 24 h; R12h/24 h/36 h: oxygen-glucose deprivation 2 h and reoxygenation 12 h/24 hrs/36 hrs. *P < 0.05 vs. Control

To elaborate the correlation between ERK pathway activation, mitochondrial dynamics and autophagy induction, we detected the expression of p-ERK, p-Drp1 and LC3II at different time points of OGD/R with immunofluorescence staining in Fig. 8A (OGD1h/2 h/3 h reoxygenation 24 h and OGD2h reoxygenation 12 h/24 hrs/36 hrs). In Fig. 8B ~ D, p-ERK, p-Drp1 and LC3II were activated by OGD/R. The expression level of p-ERK increased with OGD time extended, the expression levels of p-Drp1 and LC3II were highest in OGD2h and reoxygenation 24 h.

### Inhibition of ERK promotes expression of mitochondrial fusion proteins by increment of Mfn2 in OGD/R-treated SH-SY5Y cells

**Fig. 9:**
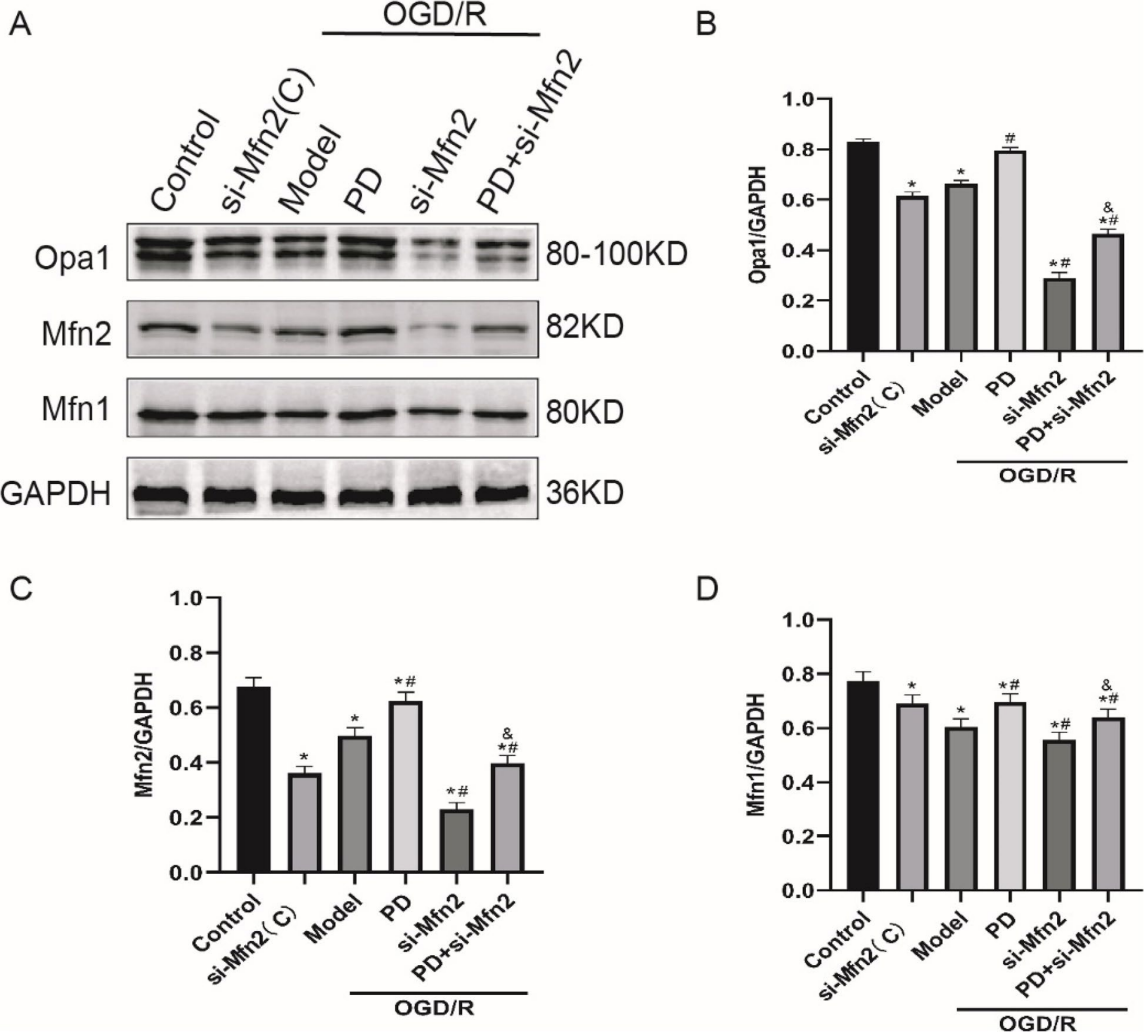
Inhibition of ERK promotes expression of mitochondrial fusion proteins by increment of Mfn2 after OGD/R in SH-SY5Y cells. **A** Representative immunoblot bands of mitochondrial fusion proteins: Opa1, Mfn2 and Mfn1; **B**-**D**, Quantitative analysis of expression of Opa1, Mfn2 and Mfn1. All data in this figure are representative of three independent repeats and are shown as mean ±SEM. si-Mfn2(C): Mfn2 si-RNA without OGD/R group; PD: PD98059 group; Mfn2: Mfn2 si-RNA group. *P < 0.05 vs. Control, #*P* < 0.05 vs. Model, &*P* < 0.05 vs. PD

Since we have determined that Drp1-depengdent mitochondrial dynamics was implicated in OGD/R-induced injury, nonetheless, mitochondrial dynamics are not only regulated by Drp1 but also by Mfn2-dependent fusion, we used Mfn2 si-RNA to further investigate the mechanisms of ERK-Mfn2-autophagy pathway in the same model. As shown in Fig. 9A ~ D, PD increased the expression levels of Opa1/Mfn2/Mfn1 (*P* < 0.05). Si-Mfn2 elicited opposite effects (*P* < 0.05) and partly reversed the PD-mediated increment of mitochondrial fusion proteins (*P* < 0.05). These results suggested that inhibition of ERK reduces the OGD/R-induced decline of mitochondrial fusion proteins partly by increment of Mfn2 expression.

### Inhibition of ERK downregulates autophagy through increasing Mfn2 in OGD/R-treated SH-SY5Y cells

**Fig. 10:**
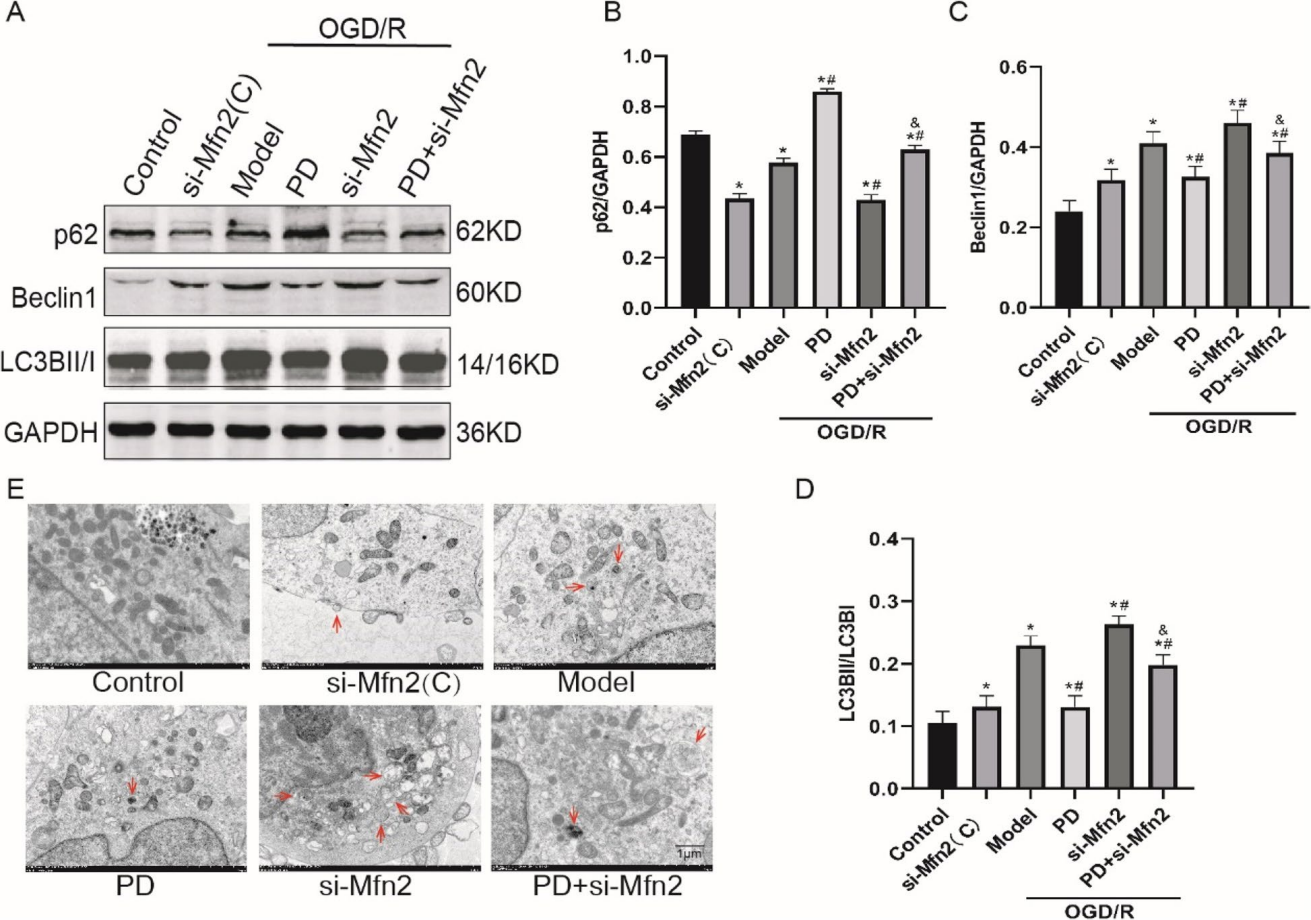
Inhibition of ERK downregulates autophagy through increasing Mfn2 in OGD/R-treated SH-SY5Y cells. **A** Representative immunoblot bands of LC3B, Beclin1 and p62. **B**-**D**, Quantitative analysis of expression of LC3B, Beclin1 and p62. All data in this figure are representative of three independent repeats and are shown as mean ±SEM. **E** The transmission electron microscopy images of autophagosomes/autolysosomes (red arrows). Scare bar: 1 μm. si-Mfn2(C): Mfn2 si-RNA without OGD/R group; PD: PD98059 group; Mfn2: Mfn2 si-RNA group. *P < 0.05 vs. Control, #*P* < 0.05 vs. Model, &*P* < 0.05 vs. PD

To explore the mechanism of ERK-Mfn2-autophagy pathway in OGD/R-treated SH-SY5Y cells, we first observed the effect of Mfn2 si-RNA on autophagy (Fig. 10A). In Fig. 10B ~ D, si-Mfn2 further aggravated OGD/R-induced excessive autophagy flux, as indicated by the increase in LC3BII/I and Beclin1 expression (*P* < 0.05), and the decrease in p62 expression (*P* < 0.05); Mfn2 si-RNA abolished the downregulation of autophagy by PD (*P* < 0.05). The changes of autophagosomes/autolysosomes in different groups (Fig. 10E) were consistent with the expression of autophagy-related proteins.

### Inhibition of ERK prevents mPTP opening and protects from OGD/R injury by increment of Mfn2 in SH-SY5Y cells

**Fig. 11:**
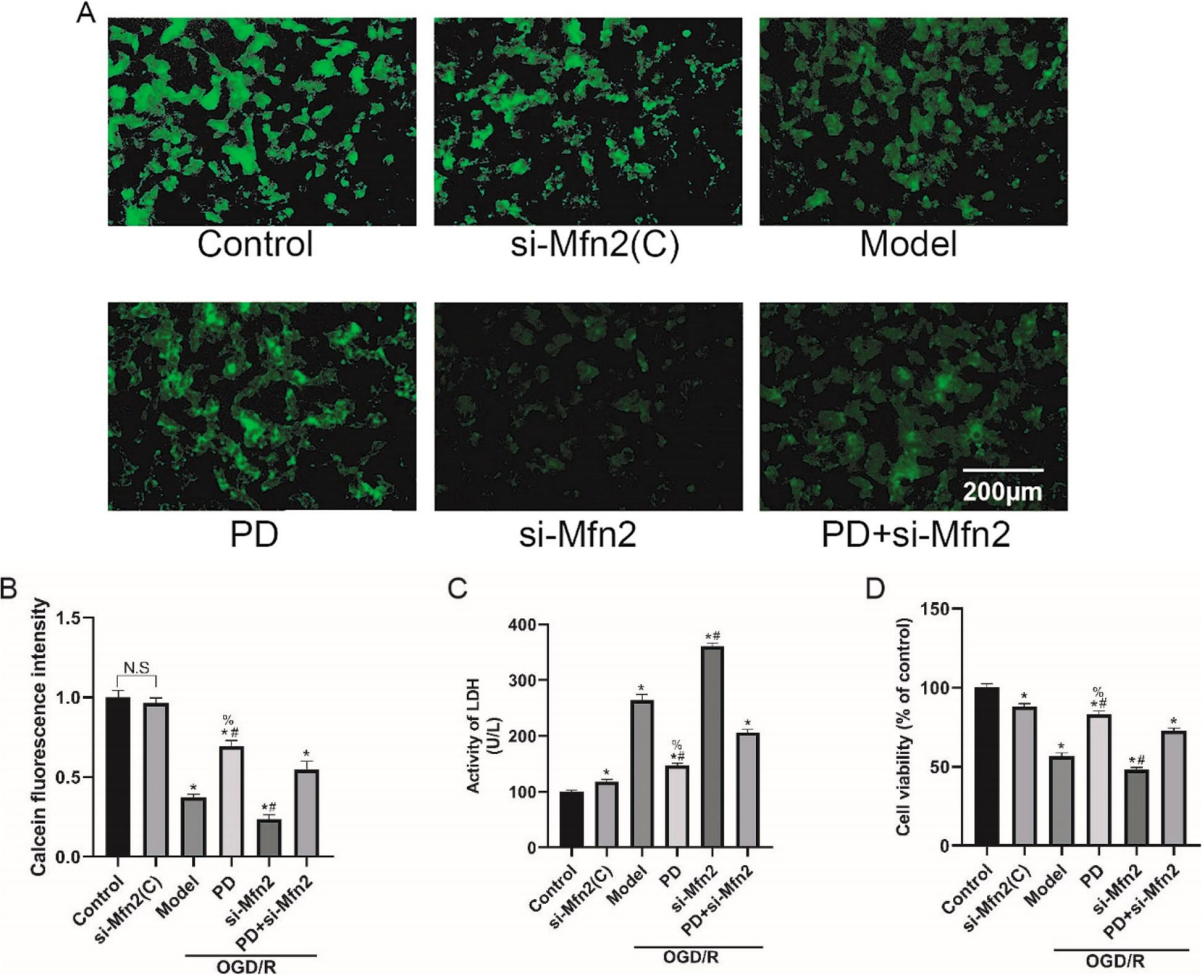
Inhibition of ERK prevents mPTP opening and protects from OGD/R injury by increment of Mfn2 in SH-SY5Y cells. A, The opening of mPTP was detected by loading with calcein AM (green fluorescence). The intensity of green fluorescence was negatively correlated with the opening of mPTP, scare bar: 200 μm. B, Quantitative analysis of calcein AM florescence intensity. C, D, LDH and CCK-8 assays were performed to detect cell injury and viability. All data in this figure are representative of three independent repeats and are shown as mean ±SEM. si-Mfn2(C): Mfn2 si-RNA without OGD/R group; PD: PD98059 group; Mfn2: Mfn2 si-RNA group. *P < 0.05 vs. Control, #*P* < 0.05 vs. Model, %*P* < 0.05 vs. PD + Mfn2

To elucidate the roles of ERK-Mfn2 in OGD/R-induced mitochondrial dysfunction and injury, we tested mPTP opening level, LDH release activity and cell viability after OGD/R in Mfn2 knockdown SH-SY5Y cells (Fig. 11A). In Fig. 11B, ablating Mfn2 resulted in mPTP opening, as shown by weaker green fluorescence (*P* < 0.05), and it also abolished the inhibitory effect of PD on mPTP opening (*P* < 0.05). In Fig. 11C and D, ablating Mfn2 aggravated OGD/R-induced injury and death, as suggested by higher LDH activity and lower cell viability (*P* < 0.05); particularly, si-Mfn2 reversed the protection of PD from OGD/R (*P* < 0.05). These results indicated that inhibition of ERK prevents mPTP opening and protects cells from OGD/R injury by restoration of Mfn2 expression in SH-SY5Y cells.

## Discussion

**Fig. 12:**
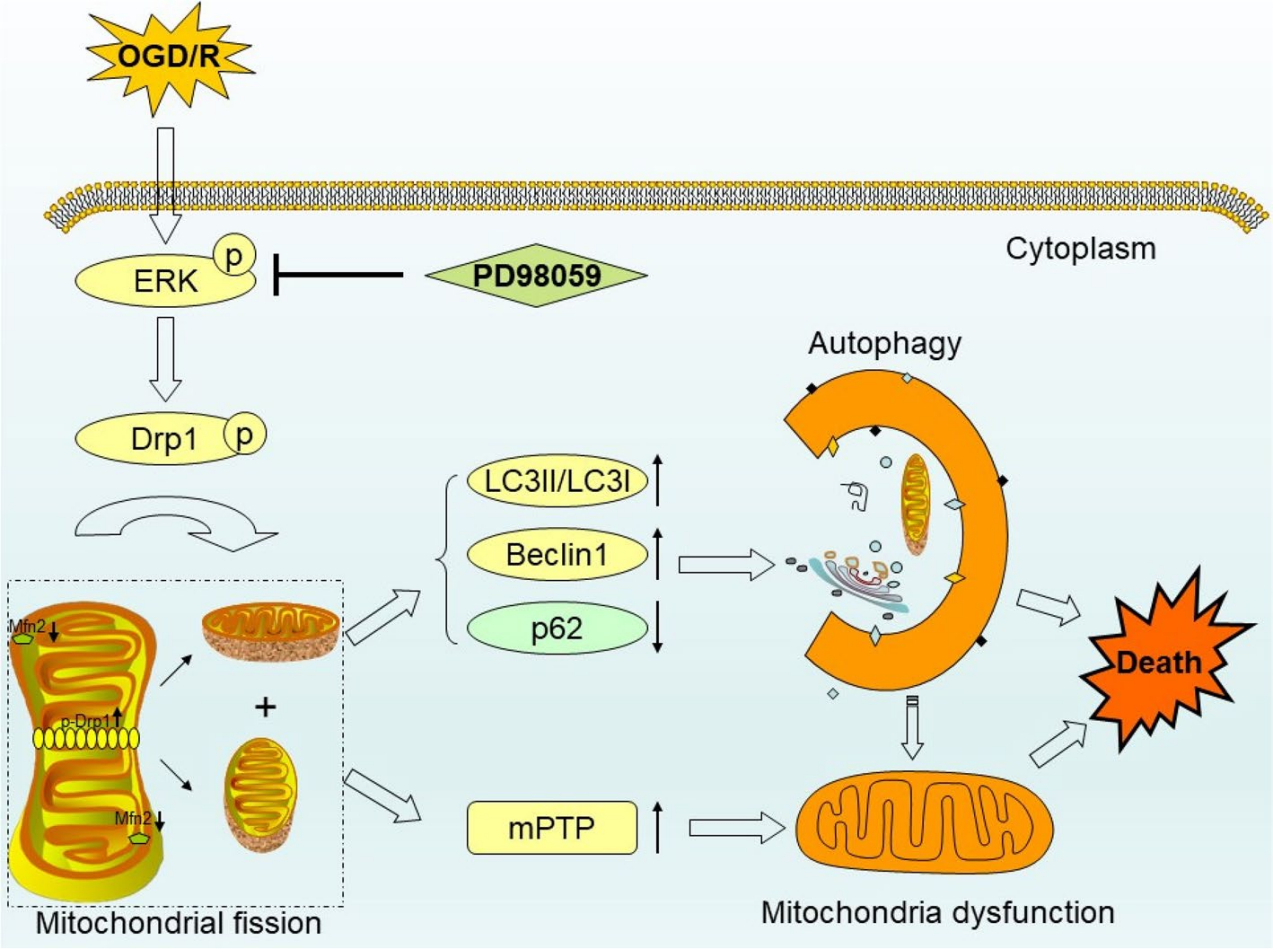
The schematic of hypothesis for ERK-mitochondrial fragmentation-autophagy axis-mediated OGD/R-induced injury in SH-SY5Y cells. Inhibition of ERK downregulates autophagy via ameliorating Drp1/Mfn2-dependent mitochondrial fragmentation, recovering mitochondrial function and reducing neuron death. OGD/R: Oxygen glucose deprivation/reoxygenation; p: Phosphorylation; ERK: Extracellular signal-regulated kinase; Drp1: Dynamin-related protein 1: Mfn2: Mitofusin2; LC3: Microtubule-associated protein light chain 3; Beclin1: BECN 1; p62: Sequestosome 1; mPTP: Mitochondrial permeability transition pore

In this study, we elucidated that ERK, mitochondrial fission and autophagy are all excessively activated and hence aggravate OGD/R-induced neuron injury; furthermore, we demonstrated that inhibition of ERK downregulates autophagy via ameliorating Drp1/Mfn2-dependent mitochondrial fragmentation, which recovers mitochondrial function and improves neuron survival (Fig. 12).

ERK protein belongs to MAPK family, regulating several vital physiological and pathological processes including ischemia/hypoxia, oxidative stress, cancer, apoptosis, etc. [22–24]. Our previous study [18] found that inhibition of ERK antagonists brain injury in the CA/CPR rat model in vivo. Similarly, the current study showed that ERK inhibition ameliorates OGD/R-induced injury and improves cell viability in SH-SY5Y cells in vitro; in contrast, activation of ERK further exacerbates cell injury and death. Similar evidences in different organs ischemia-reperfusion models have also been reported. Alessandrini et al. [25] showed that PD reduces cerebral infarction volume by 55% at 24 h and 36% at 72 h after MCAO. Ye et al. [26] confirmed that ERK activation drives cardiomyocytes apoptosis in the rat myocardial ischemia-reperfusion model. However, some studies [27, 28] supported the neuroprotection of ERK in CIRI. The discrepancy may be due to the intricate extensive molecular signal pathways in the downstream of ERK. The additive effect of multiple pathway is determined in response to specific pathological conditions, cell types, experimental procedures, and observation time points.

The autophagic flux in our experiment was monitored using the expression level of LC3, Beclin1 and p62. Beclin1 recruits a variety of autophagy proteins by combining with other autophagy related proteins to form the Beclin1-vacuolar protein sorting 34 (Vps34) complex at the initial stage of autophagy, which is essential for the autophagosome formation [29]. LC3 is a key constituent of autophagosome, where LC3I converts to LC3II. The ratio of LC3II/I indicates the activity of autophagosome formation [30]. P62 has a LC3-interacting domain and is degraded within the autolysosome, triggering the process of autophagy flux, therefore, the accumulation of p62 indicates an impaired autophagy flux [31]. In our study, we observed PD reduces autophagy flux as evidenced by decreased LC3II/I and Beclin1, increased p62 accumulation. This was consistent with our previous study in vivo. What’s more, like 3-MA, PD alleviates OGD/R-induced injury and death, while rapamycin exerts opposite effects. Since overactivated autophagy contributes to degradation of numerous crucial proteins and organelles, and then induces apoptosis, necrosis or directly results in type II programmed cell death [32, 33]. Therefore, inhibition of ERK at least partially protects cells from OGD/R injury via mitigating excessive autophagy. This result is consistent with an earlier study [34] which suggested that the autophagic flux is increased after OGD exposure in the primary mouse neuron cells, and downregulation of autophagic flux by co-culture of the neuron with adipose-derived mesenchymal stem cells significantly reduces neuron death. However, some scholars revealed that autophagy helps cells decompose damaged proteins and organelles to provide nutrients for cell survival under pathological circumstances. Zha et al. [35] suggested that activation of autophagy by rapamycin protects neurons from CIRI in vitro and in vivo via inhibiting neuroinflammation. The discrepancy possibly results from the different level of autophagy upon disparate experimental protocols and models. Autophagy is a double-edged sword and a proper level of autophagy is vital to maintain intracellular homeostasis. Insufficient and excessive autophagy are both maladaptive to cell survive. In our study, PD and 3-MA ameliorated cell death by downregulating excessively activated autophagy to the appropriate level. While in Zha et al.’s study, incomplete autophagy by OGD/R and MCAO failed to antagonist cell death.

In this experiment, si-RNA was transfected into cells to inhibit the translation of Drp1 and Mfn2. We found that Drp1 si-RNA reduced the expression of both Drp1 and p-Drp1, interestingly, the reduction of p-Drp1 was much more significant. The probable reason is either that the balance of p-Drp1/Drp1 was broken by Drp1 si-RNA transfection, resulting in the dephosphorylation of p-Drp1, or that the si-RNA may be more likely to bind to p-Drp1 due to its spatial structure and charge characteristic. Of course, it needs more future studies to clarify. The relevance of mitochondrial dynamics proteins was confirmed by our data, showing that ablating Mfn2 further decreased Mfn1 and Opa1 expression, increased p-Drp1 expression after OGD/R; furthermore, ablating Mfn2 abolished the effect of PD on promoting Mfn1 and Opa1, reducing p-Drp1. Therefore, Mfn2 may be a critical protein regulating mitochondrial dynamic balance and mediates the effect of ERK on mitochondrial fragmentation. Excessive mitochondrial fission induced by the increment of Drp1S616 phosphorylation and decrement of Mfn2 intensifies mitochondrial dysfunction and cell death in CIRI. Flippo et al. [36] found Drp1 inactivation attenuates CIRI-induced mitochondrial respiratory dysfunction, Ca^2+^ overload and neuronal excitatory toxicity in mice brain. This is in line with our current study. We documented alleviation of mitochondrial fragmentation by Drp1 knockdown prevents mPTP opening and protects cells from OGD/R-induced injury, whereas aggravation of mitochondrial fragmentation by Drp1^S616E^ overexpression or Mfn2 knockdown elicits opposite effects.

The effect of ERK on mitochondrial dynamics has been validated in some studies. Young et al. [37] demonstrated inhibition of deacetylase sirtuin 2 (SIRT2) activates Drp1 via ERK. Macrophage stimulating 1 (Mst1) enhances IL24-based anti-tumor via inactivating ERK-Mfn2 pathway [38]. We also clarified inhibition of ERK alleviates Drp1/Mfn2-dependent mitochondrial fission. Mitochondrial dynamics plays an important role in autophagy. The size of the mitochondria is about 2000–10,000 nm, which is much larger than autophagosome (500–1500 nm). The upregulation of mitochondrial fragmentation via activating Drp1 or inhibiting Mfn2 obviously reduces mitochondrial size and thus promotes autophagy. Recent studies found that when Drp1 is transferred to mitochondria, it could release Beclin1 and interact with LC3 to activate autophagy [39, 40]. In addition, Mfn2 localizing on the mitochondrial-associated endoplasmic reticulum membrane (MAM) also participates in autophagy via interacting with autophagy-related proteins [41].

Previously, we observed that mitochondrial and autophagy were both reduced by PD in neuron after CA/CPR. Currently, we found that Drp1^S616E^ overexpression and Mfn2 knockdown further increase OGD/R-induced autophagy flux and autophagosomes, while Drp1 knockdown and PD has the opposite effects, demonstrating that Drp1/Mfn2-dependent mitochondrial dynamics and ERK could determine the cellular autophagic level in OGD/R. Of note, we observed that the autophagy level in PD + Drp1 knockdown group is significantly lower than that in single groups, indicating the effects of ERK and mitochondrial fragmentation on autophagy are not completely via the same pathway. Multiple immunofluorescence staining also displayed the co-expression of p-ERK, p-Drp1S616 and LC3. Based on these evidences, we speculated ERK-mitochondrial dynamics-autophagy axis implicated in OGD/R-induced injury. To verify this conclusion, we used Drp1/Mfn2 transfection and found that Drp1^S616E^ overexpression and Mfn2 si-RNA partly abolished the inhibitory effect to autophagy by PD. Similar to our study, the ERK-mitochondrial dynamics-autophagy pathway has been reported involved in heart failure [42], pulmonary arterial hypertension [43], the chemotherapy resistance of colorectal cancer cells [20], etc. However, there are few reports on this molecular signaling pathway in CIRI.

There are still some limitations in this study: Firstly, the experimental model was derived from human nerve cells cultured in vitro. The species source is more consistent with the disease objects we aimed to study and the interference of many complicated factors in animal experiments is excluded, but the conclusions need to be further verified in animal models. Secondly, we only detect autophagy without further investigating mitochondrial autophagy (mitophagy) and its pathway. Since mitochondrial dynamics imbalance is inclined to stimulating mitophagy, further work is required to explore the mechanism of ERK-mitochondrial dynamics-mitophagy axis on CIRI.

In summary, our study highlighted that the ERK-mitochondrial dynamics-autophagy axis is involved in the pathological mechanism in OGD/R-induced injury and demonstrated that inhibition of ERK downregulates autophagy via reducing Drp1/Mfn2-dependent mitochondrial fragmentation to antagonize mitochondrial dysfunction and promote cell survival.

### Supplementary Information

**Additional file 1.**

**Additional file 2.**

## Data Availability

All data supporting the findings of the study are included within this article.
